# The Emerging Roles of Endoplasmic Reticulum Stress in Balancing Immunity and Tolerance in Health and Diseases: Mechanisms and Opportunities

**DOI:** 10.3389/fimmu.2019.03154

**Published:** 2020-02-11

**Authors:** Anqi Li, No-Joon Song, Brian P. Riesenberg, Zihai Li

**Affiliations:** ^1^College of Medicine, The Ohio State University, Columbus, OH, United States; ^2^The James Comprehensive Cancer Center, Pelotonia Institute for Immuno-Oncology, The Ohio State University, Columbus, OH, United States; ^3^Division of Medical Oncology, Department of Medicine, The Ohio State University, Columbus, OH, United States

**Keywords:** endoplasmic reticulum stress, immunity, diseases, therapeutics, inhibitors

## Abstract

The endoplasmic reticulum (ER) is an organelle equipped with mechanisms for proper protein folding, trafficking, and degradation to maintain protein homeostasis in the secretory pathway. As a defense mechanism, perturbation of ER proteostasis by ER stress agents activates a cascade of signaling pathways from the ER to the nucleus known as unfolded protein response (UPR). The primary goal of UPR is to induce transcriptional and translational programs to restore ER homeostasis for cell survival. As such, defects in UPR signaling have been implicated as a key contributor to multiple diseases including metabolic diseases, degenerative diseases, inflammatory disorders, and cancer. Growing evidence support the critical role of ER stress in regulating the fate as well as the magnitude of the immune response. Moreover, the availability of multiple UPR pharmacological inhibitors raises the hope that targeting UPR can be a new strategy for immune modulation and immunotherapy of diseases. This paper reviews the principal mechanisms by which ER stress affects immune cell biology and function, with a focus of discussion on UPR-associated immunopathology and the development of potential ER stress-targeted therapeutics.

## Introduction: ER Stress, Immunity, and Diseases

The endoplasmic reticulum (ER) is an essential organelle which participates in protein quality control in the secretory pathway of all eukaryotic cells (1, 2). ER homeostasis is critical for controlling various intracellular physiological functions including protein folding, calcium homeostasis, lipid metabolism, cell differentiation, and protein translocation (3, 4). However, under certain circumstances such as nutrient deprivation, hypoxia, acid–base imbalance, and accumulation of reactive oxygen species (ROS), the capacity of ER function can be exceeded, resulting in the accumulation of misfolded proteins (5–9). The unfolded protein response (UPR) then ensues, which is one of the evolutionally conserved and protective mechanisms of the ER to resolve stress and dysfunction. Molecularly, the UPR can initiate several intracellular responses. First, the overall protein synthesis is attenuated while favoring the upregulation of protein chaperones to promote protein processing and refolding. In the event when increased chaperones cannot meet the folding/refolding demand, the UPR triggers protein catabolism via the ER-associated degradation pathway (10). The UPR mechanism also gears toward expanding the physical space of ER by increasing the synthesis of ER membranes through promoting lipid metabolism (11). Finally, if these mechanisms fail to reverse chronic ER stress, the UPR will induce cell death via apoptosis.

Diseases like autoimmunity and cancer are regarded as consequence of unbalanced immune response. Given that maintaining immune cell homeostasis is important for balancing effector and regulatory function, emerging evidence suggests ER stress, specifically in the immune compartment, participates in various pathologies including neurodegeneration, inflammation, metabolic disorders, and infectious diseases. In addition, ER stress hinders antitumor immunity through regulation of immunosuppressive cells including type 2 macrophages (M2), myeloid-derived suppressor cell (MDSCs), tolerogenic dendritic cells (DCs), and others (9, 12–15). Taken together, these studies suggest that controlling balance of ER stress can serve as important therapeutic strategy for multiple diseases (9, 16).

In this paper, we will examine the link between ER stress of the immune cells and diseases. Particularly, we will provide evidence linking the effector molecules of the UPR to their roles in regulating immunity. We will also discuss the therapeutic potential of known small molecule inhibitors targeting the UPR in treatment of diseases such as cancer. We call for further studies of UPR pathways and their inhibitors for immune modulation in clinically relevant models, before the promise of UPR targeting as an immunotherapeutic strategy can be utilized.

## Roles of Major UPR Sensors in Cellular Homeostasis and Diseases

### IRE1

**Figure 1:**
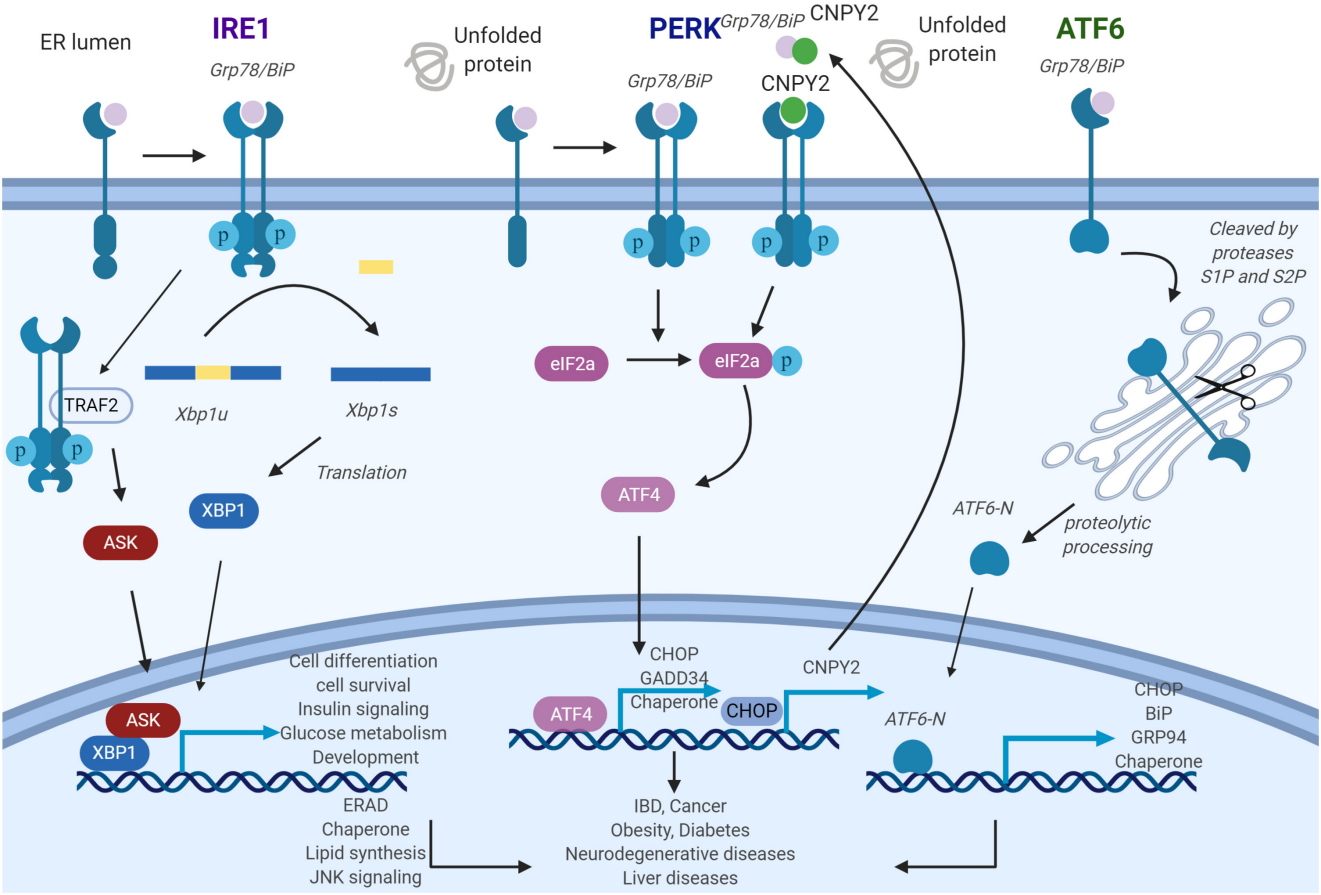
General roles of unfolded protein response (UPR) pathways endoplasmic reticulum (ER) stress sensors inositol-requiring enzyme 1 (IRE1), PKR-like ER kinase (PERK), and activating transcription factor 6 (ATF6) deliver ER stress signals from the ER lumen into the cytosol. IRE1 pathway: ER stress induces IRE1 oligomerization and autophosphorylation, then the splicing of XBP1 is triggered by activated IRE1. As a transcription factor, X-box binding proteins 1 (XBP1s) activate UPR-related genes. PERK pathway: The activated PERK phosphorylates eIF2a and further stimulates ATF4, which will regulate its target gene expression. Canopy homolog 2 (CNPY2) could dissociate from Grp78 and then promote PERK autokinase activity. Increased translation of CAAT/enhancer-binding protein homologous protein (CHOP) activates CNPY2 promoter and further elevates CNPY2 expression. ATF6 pathway: ATF6 is cleaved by proteases S1P and S2P to produce ATF6-N. ATF6-N then migrates to the nucleus to initiate the transcription of its target genes. IRE1-XBP1, PERK, and ATF6 pathways, if protracted, can contribute to the development of various diseases. Figure was made with Biorender.

ER stress signals are transduced largely by three conserved transmembrane proteins on the ER membrane known as ER stress sensors: inositol-requiring enzyme 1 (IRE1), PKR-like ER kinase (PERK), and activating transcription factor 6 (ATF6). The discussions of each of the sensors ensue. Inositol-requiring enzyme 1 (IRE1) is composed of two isoforms: IRE1α and IRE1β (17). IRE1α gene knockout mouse is embryonically lethal while the IRE1β knockout does not show severe phenotypic abnormality, indicating non-redundant functional roles. Indeed, the expression pattern of the isoforms appears to validate this as IRE1α is ubiquitously present while IRE1β expression is limited to the pulmonary mucosal epithelium and the gastrointestinal tract (18, 19). IRE1 is a type I transmembrane protein with ER-luminal sensor for peptide recognition and serine/threonine kinase activity (Figure 1). IRE1 also displays endoribonuclease activity in the C-terminal domain (20). Accumulation of unfolded proteins causes the dissociation of the immunoglobulin binding protein (BiP) from the luminal domain of IRE1 which triggers immediate IRE1 oligomerization and autophosphorylation of its kinase domain (21, 22). As a response to this conformational change, IRE1 RNase domain is activated which induces the unconventional cytosolic splicing of *X-box binding protein 1* (*XBP1)* messenger RNA (mRNA) to generate an alternatively spliced XBP1 known as XBP1s with shifting of the 3′ open reading frame (22). To restore ER homeostasis, XBP1s stimulates the transcription of various target genes including protein folding chaperones and the effector molecules in the ER-associated degradation pathway (23). Besides maintaining homeostasis, XBP1s also participates in multiple cellular signaling pathways such as cell differentiation, survival, insulin signaling, glucose metabolism, and development (14, 18, 24–27). Recently, it was discovered that the activation of RNase activity not only increases unconventional splicing of *XBP1* but also targets multiple other transcripts through a distinct mechanism called regulated IRE1-dependent decay (RIDD) (28). Systemic analysis of RNase activity of wild type (WT) and IRE mutant revealed multiple binding substrates (29, 30). RIDD selectively cleaves mRNAs encoding proteins involved in protein folding and ER stress regulation and chronic activation of RIDD signaling promotes cell death mechanism (23, 31). In addition to endonuclease activity, IRE1 activates JNK signaling through direct interaction of IRE1 to tumor necrosis factor (TNF) receptor associated factor 2 (TRAF2) (32). This IRE1-TRAF2 complex recruits and activates apoptosis signal-regulating kinase 1 (ASK1), leading to activation of c-Jun N-terminal kinase (JNK) pathway which ultimately triggers cell death (33).

Numerous studies have revealed importance of ER stress response in immunity and inflammation. One of the most well-studied ER stress related inflammatory disease is inflammatory bowel disease (IBD) (34, 35). IBD is a human chronic inflammatory disorder of the gut with distinct clinical manifestation and pathology but complicated underlying pathogenesis. Studies have shown that IRE1-XBP1 pathway protects mice from experimental model of IBD (36). IRE1β, a specific isoform of IRE1, is expressed in epithelial cells of the gastrointestinal tract. IRE1β deficient mice were more susceptible to dextran sulfate sodium (DSS) induced colitis than WT controls (37). In addition, XBP1, the downstream molecule of IRE1, behaves oppositely in the mouse colitis model. The mice with a XBP1 deficiency in the epithelial cells displayed a spontaneous enteritis and Paneth cell dysfunction which implicates the important role of ER stress in IBD. In this study, authors also provided evidences that single nucleotide polymorphisms (SNPs) in XBP1 gene locus are positively associated with human IBD (38). Rather than IBD, XBP1 also plays a role in inflammation in different cell types such as macrophages and DCs. ER stress increases cytokine productions including interleukin-1 (IL-1), IL-6, IL-8, TNFα, and monocyte chemoattractant protein 1 (MCP1) (39–41). Tumor microenvironment (TME), a chronically inflamed condition, is characterized by high degree of ER stress. Besides modulating cancer cell function intrinsically, IRE1 profoundly regulates immune cells in the TME, which will be discussed later.

Apart from inflammatory regulation, IRE1 pathway has also been implicated in metabolic diseases including obesity and diabetes (42). Using several well-established mouse obesity models such as high fat diet (HFD) induced obese mouse model and leptin deficient mouse model, it was found that obesity is associated with increased expression of phosphorylated IRE1, PERK, and JNK in adipose tissue and the liver. The XBP1 deficient mice display impaired glucose homeostasis when compared to WT controls. Mechanistically, XBP1s suppresses insulin receptor signaling through hyperactivation of JNK and phosphorylation of insulin receptor substate-1 (IRS-1) (43). IRE1 signaling regulates pancreatic β cell damage under prolonged or excess ER stress which leads to the development of diabetes (44). Moreover, high glucose mediated chronic ER stress in β cell induces activation of IRE1 signaling, leading to the degradation of proinsulin mRNA (45). In line with these observations, pancreatic β cell specific XBP1 deficient model shows glucose intolerance due to decreased insulin secretion, implicating crucial role of ER stress in metabolic diseases (46).

### PERK

Protein kinase RNA-like ER kinase (PERK), a type 1 transmembrane protein with serine/threonine kinase activity in its C-terminus, recognizes the accumulation of misfolded proteins by its luminal domain sensor (20). Activation of PERK is initiated by dissociation of BiP from its luminal domain resulting in its oligomerization and autophosphorylation (47). Active PERK phosphorylates eIF2α, which results in a reduction in general protein synthesis thereby decreasing the load of proteins entering the ER. This rapid response serves as prosurvival mechanism (17). Remarkably, under these circumstances, some transcripts such as activating transcription factor 4 (ATF4) are translated more efficiently. This is due to a change in the reading frame within *ATF4*, causing its induction which further stimulates the transcription of downstream UPR target genes, including CAAT/enhancer-binding protein homologous protein (CHOP) and growth arrest and DNA damage-inducible 34 (GADD34) (20, 48, 49). CHOP activates proapoptotic genes while GADD34 dephosphorylates eukaryotic translation initiation factor 2 α (eIF2α) thereby creating a negative feedback loop to restore protein synthesis and protein load into ER (50, 51) (Figure 1). Thus, PERK activates a prosurvival mechanism at first, but switches to proapoptotic mechanism under prolonged ER stress by regulating CHOP and GADD34. Recently, our group reported that ER luminal protein canopy homolog 2 (CNPY2) is detached from BiP under ER stress. Free CNPY2 then activates PERK–CHOP pathway and enhances UPR signaling. Our finding thus revealed a new mechanism of UPR initiation especially for the PERK branch: PERK activation can be triggered by both BiP dissociation and its direct binding to CNPY2 (52). Not surprisingly, CNPY2 deletion protects mice from non-alcoholic fatty liver disease (NAFLD). The roles of CNPY2 in inflammation and immune responses are under active investigation.

PERK pathway has been implicated in various diseases especially in neurodegenerative diseases and metabolic diseases. ER stress delays degradation of tau protein and causes hyperphosphorylation of tau, which in turn further amplify UPR, creating a vicious cycle (53). Another major protein in neurodegenerative disease is amyloid β. Amyloid β oligomers or fibrils trigger PERK pathway in neuronal cells, and further investigation revealed that Ca^2+^ as a possible mediator of this action (54). In the metabolic diseases, PERK phosphorylates eIF2a, which induces ATF4 translocation and inhibits translation of cells (42). PERK pathway also manipulates IKKβ pathways in adipocytes and promotes inflammatory cytokine productions (55). In addition, activation of PERK in mouse brain astrocytes was shown to accelerate brain inflammation through increased IL-6 expression and this process was regulated by JAK/STAT3 signaling pathway (56). PERK plays important role in regulating immune cells, which will be the subject of later discussion.

### ATF6

Activating transcription factor 6 (ATF6) is a leucine zipper transcription factor and a type II ER transmembrane protein (57), consisting of ATF6α and ATF6β. ATF6 has been shown to have an essential role in development, as ATF6 null mice are embryonically lethal. Interestingly, genetic deletion of only the α or β subunit of ATF6 does not affect viability, suggesting overlapping or compensatory roles for the protein subunits (58). In response to the accumulation of mis-folded proteins in the ER, BiP dissociates from ATF6 allowing for BiP's interaction with mis-folded proteins (47). Thereafter, free ATF6 translocates from the ER to the Golgi where it undergoes cleavage mediated by two different proteases. The cleaved N-terminal cytosolic domain of ATF6 then migrates into nucleus where it activates target genes including BiP, Grp94, and CHOP leading to improved protein folding activity in the ER (23). Additionally, ATF6 has also been shown to regulate micro-RNA expression level (59). The mechanisms regulating ATF6 translocation from the ER to the Golgi, either alone or via interaction with a shuttle protein, remains unclear (Figure 1).

High ATF6 expression correlates with poor prognosis of colorectal cancer patients which is consistent with the work in the mouse model. Mouse model with the epithelial-cell-specific overexpression of activated ATF6 spontaneously developed colon adenomas (60). ATF6 also contributes to chemotherapy resistance by regulating mTOR signaling *in vivo* (61). Similar to other ER stress factors, ATF6 null mice are prone to develop hyperlipidemia and insulin resistance (62). ATF6 knockout mouse also exhibit liver dysfunctions and steatosis (63).

## Immunological Impacts of ER Stress

**Figure 2:**
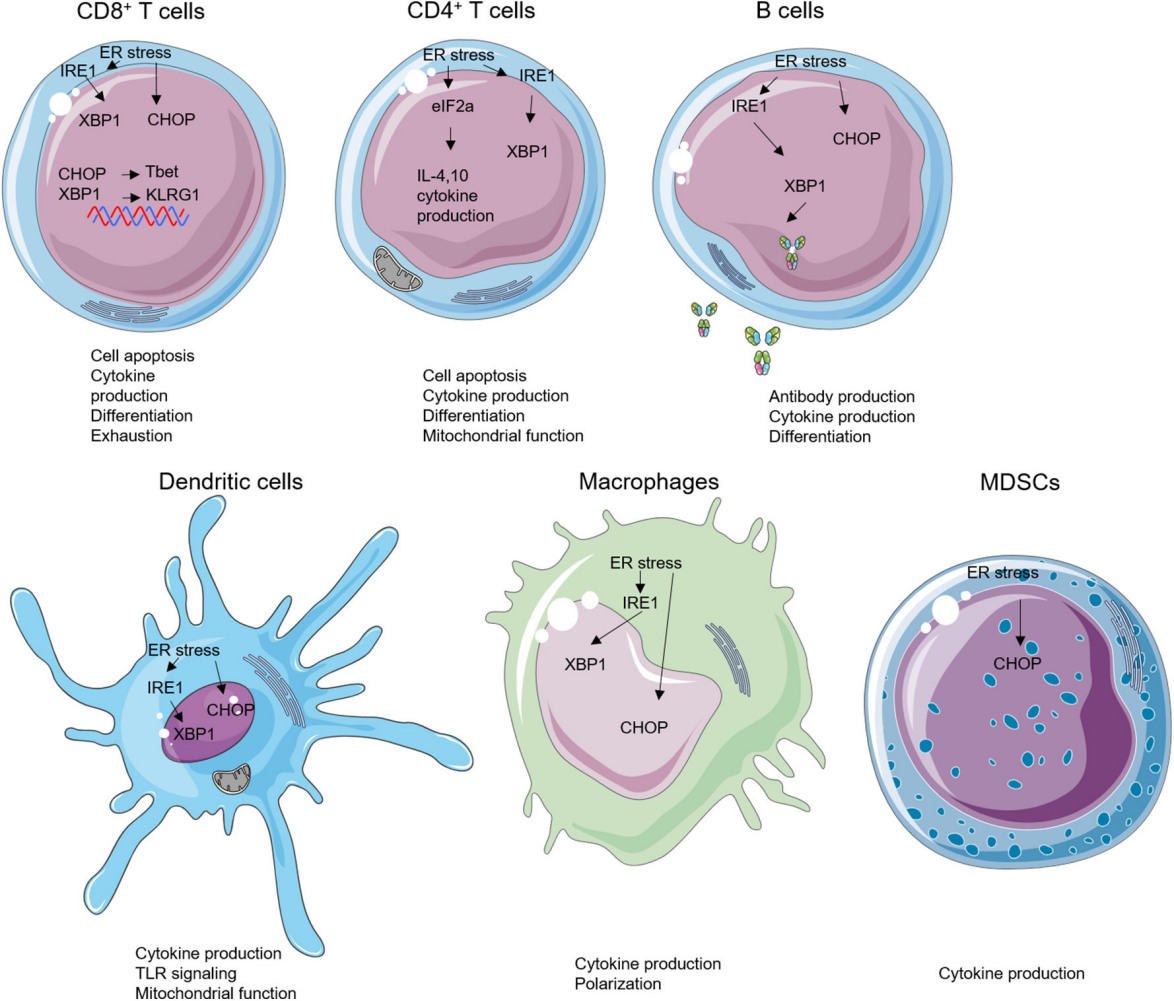
Endoplasmic reticulum (ER) stress and unfolded protein response (UPR) effectors in immune cells. ER stress can modulate the biology of various subsets of immune cells such as cell apoptosis, cytokine production, cell differentiation, antibody production, mitochondrial function, and Toll-like receptor (TLR) signaling.

**Figure 3:**
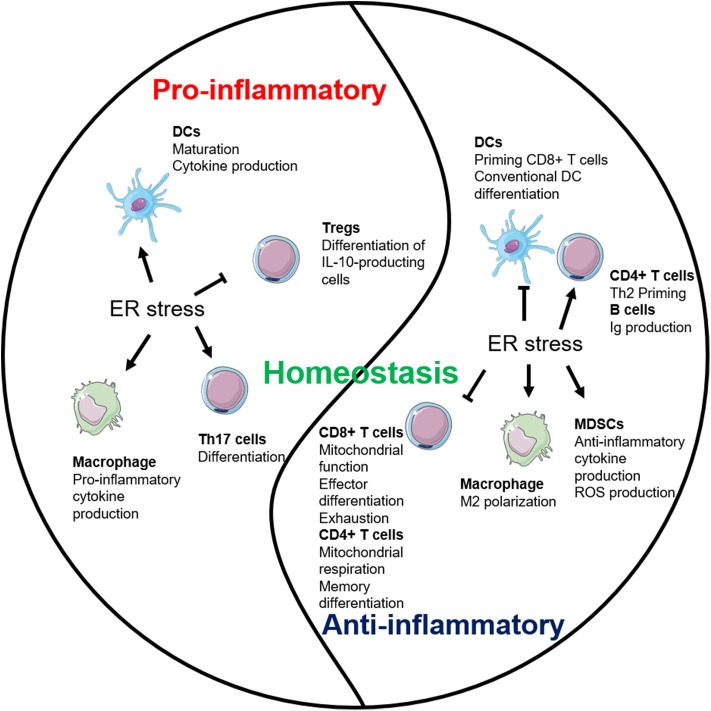
Endoplasmic reticulum (ER) stress plays multifaceted roles in inflammation. ER stress establishes the homeostatic environment of both pro- and anti-inflammation through regulating major immune cells.

The ER stress plays critical roles in controlling various intracellular physiological functions including protein folding, calcium homeostasis, lipid metabolism, cell differentiation, and protein translocation (20). Thus, malfunction of the ER stress response has been linked, not surprisingly, to dysregulation of the innate and adaptive immune response. Recent work has demonstrated that the UPR sensors are involved in regulating the development, differentiation, activation, cytokine production, and apoptosis of multiple immune cell types including T cells, B cells, DCs, macrophages, and MDSCs (Figures 2, 3). As such, the emerging roles of UPR effectors in the immune compartment raise a possibility of targeting UPRs in the management of a number of immune disorders including cancer. For ease of information flow, we will discuss the roles of UPR in each individual immune cell types, although the impact of UPR on the overall immune response at organismal level is obviously mediated at the multiple cell level.

### CD8^+^ T Cells

Transformed tumor cells or cells infected with intracellular pathogens can be recognized and eliminated by cytotoxic CD8^+^ T cells via T-cell receptor engagement and subsequent activation and cytokine secretion. ER stress regulates differentiation, cytokine production, exhaustion, and apoptosis of CD8^+^ T cells. Following acute viral or bacterial infection, expansion of antigen-specific CD8^+^ T cell population occurs, and this process increases levels of spliced XBP1 mRNA. Further investigation revealed that the enhanced XBP1 splicing was not only enriched but also required for the expression of killer cell lectin-like receptor G1 (KLRG1) in CD8^+^ T cells (64). Apart from regulating cell differentiation, ER stress–XBP1 pathway is required for cholesterol-induced CD8^+^ T-cell exhaustion and expression of inhibitory molecules such as programmed cell death protein-1 and 2B4. In the murine B16 melanoma TME, high level of cholesterols disrupts the lipid metabolism and stimulates XBP1s expression in CD8^+^ T cells. Furthermore, inhibiting XBP1s or reducing cholesterol enhances antitumor effect of CD8^+^ T cells *in vivo* (65). In addition, Cao et al. demonstrated that CHOP negatively regulates effector and mitochondrial function of CD8^+^ T cells. Accordingly, deletion of CHOP in CD8^+^ T cells improves the antitumor immune response through direct increase of T-bet transcription (66). GRP78 (BiP) also plays an important role in regulating inflammatory cytokine productions in the Crohn's disease-like ileitis. This ER-stress-associated UPR is essential for granzyme B-dependent CD8αβ^+^ T-cell cytotoxicity (67).

ER stress has been linked to CD8^+^ T-cell metabolic regulation and fitness in several disease settings (68, 69). Indeed, mitochondrial function is essential for the CD8^+^ T cells (68). Genetic and pharmacological inhibition of PERK in CD8^+^ T cells abrogates mitochondrial ROS generation in programmed cell death protein-1+ CD8^+^ tumor-infiltrating lymphocytes (TILs), which bolsters CD8^+^ tumor-infiltrating lymphocyte viability and enhances antitumor immunity (70). Multiple ER stress sensors such as CHOP, phosphorylated eIF2α, and GRP78 induce T-cell apoptosis, implicating that ER stress could block apoptosis and increase T-cell persistence (71–73). However, the mechanisms explaining the relationship between UPR pathways and CD8^+^ T cells have not been precisely demonstrated in many diseases especially in cancers. The impact of pharmacological targeting of ER stress on CD8^+^ T cells in the tumor microenvironment requires further investigation (Table 1).

**Table 1:** Role of unfolded protein response (UPR) effectors in specific immune cell populations.

	**IRE1**	**PERK**	**ATF6**
CD8+ T cells	Increases T-cell differentiation Induces T-cell exhaustion (64, 65)	Negative regulator of effector T cell (66, 70)	Increase cytokine production (67)
CD4+ T cells	Increases IL-4 production Increases T-cell differentiation Inhibition blocks IL-5 production (74–77)	ATF4 positively regulates CD4^+^ T cells glycolysis, glutaminolysis, and oxidative phosphorylation (78).	Unknown
B cells	Required in B-cell lymphopoiesis Activated during B-cell development (79, 80)	Knockout does not affect antibody secretion (81)	Unknown
DCs	Does not regulate conventional splenic type 1 DCs survival, but impairs survival of mucosal DCs (82)	Increase IL-23 expression (83)	Unknown
Macrophage	Suppresses alternative activation Regulates cytokine production (15, 84, 85)	Knockdown increases M2 polarization (85, 86)	Regulates TLR response (87)
MDSCs	ER stress response drives TRAIL-R upregulation (88, 89)	ER stress response drives TRAIL-R upregulation (88)	Unknown
NK	Activate immune response (90)	Unknown	Unknown
Platelets	Proplatelet formation in megakaryocytes (91)	Unknown	Unknown

### CD4^+^ T Cells

Naive CD4^+^ T cells can differentiate into various subsets of T helper (Th) cells, including Th1, Th2, Th17, and regulatory T cells (Tregs) under different conditions (92, 93). ER stress plays an important role in regulating differentiation, plasticity, effector function, and apoptosis of CD4^+^ T cells. The activation of ER stress via phosphorylation of eIF2α occurs in the process of Th2 activation and differentiation, and, in turn, the accumulation of phosphorylated eIF2α enhances the release of IL-4 during Th2 priming (94). Cytokines in the immediate milieu during the CD4^+^ T-cell activation are largely responsible for the fate of Th differentiation. Transforming growth factor β is essential for Th17 and Treg differentiation and plasticity (95). However, ER stress can regulate the plasticity of Th17 and Tregs through a transforming growth factor β-independent mechanism. XBP1s activity promotes Th17 differentiation and has a critical function in promoting experimental autoimmune encephalomyelitis (EAE) in mice. Genetical and pharmacological inhibition of XBP1s reduces onset of EAE by reducing interferon gamma (IFNγ) production from Th17 (96). PERK pathway regulator ATF4 enhances CD4^+^ T cells glycolysis and modulates mTORC1 activation. The ATF4-deficient CD4^+^ T cells are prone to differentiate into Th17 cells instead of Th1 in the EAE model (78). ER stress induces Treg plasticity as well. The canonical stressor, thapsigargin (Tg), enhances *Il10* transcription *in vitro*. Inhibition of eIF2α dephosphorylation limits IL-10 transcription, suggesting that eIF2α phosphorylation suppresses the differentiation of IL-10-producing Tregs (97).

Our group showed that ER stress is important for CD4^+^ T-cell activation and its function. Gp96 (also known as GRP94), an ER molecular chaperone, is upregulated by ER stress and plays multiple roles in immunological activities (98). In addition, our group found that gp96 modulates cytosolic Ca^2+^ mobilization upon TCR engagement resulting in changes to activation induced glycolysis. Targeting gp96 in CD4^+^ T cells increased population of CD62L^high^CD44^low^ cells leading to enhanced antitumor immunity (99). The IRE1α-XBP1 pathway in CD4^+^ T cells has also been implicated in antitumor immunity in ovarian cancer. Mechanistically, the induction of XBP1s limits the influx of glutamine, which is necessary for sustaining mitochondrial respiration in CD4^+^ T cells under glucose-deprived conditions which reflects TME (100). Aside from the roles of UPR in effector T-cell differentiation and function, ER stress also regulates T-cell autophagy and apoptosis. ER chaperone GRP78 regulates autophagy of T cells in lupus patients, and the induction of ER stress in T cells is proven by CHOP overexpression. Furthermore, ER-stress-induced ROS promotes human T-cell apoptosis and dysfunction (101, 102). Besides all these roles, CD4^+^ T cells can also be instrumental in provoking neuroinflammation. CD4^+^ T-cell infiltration into brain can increase inflammation in certain cases including multiple sclerosis and Parkinson's disease. However, CD4^+^ T cells can also play neuroprotective role against infection, stroke, and neurodegenerative diseases. This dual role of CD4^+^ T cells in brain inflammation with ER stress needs further investigation (103–105). These findings indicate the important roles of UPR in regulating CD4^+^ T cells in a variety of diseases and provide the preclinical evidence for future clinical application (Table 1).

### B Cells

The physiological importance of UPR in B-cell differentiation and function has been demonstrated by multiple studies. The UPR effectors are elevated in B cells during differentiation into plasma cells and are required for efficient antibody production (24, 25, 106–108). Specifically, XBP1s induce UPR in the plasma cells and facilitate immunoglobulin synthesis (108). XBP1-deficient B cells can develop and be activated normally, but they failed to produce immunoglobulins (24, 109). These findings indicate that XBP1 and its downstream molecules regulate B-cell differentiation and immunoglobulin production. B-lymphocyte-induced maturation protein-1, which transcriptionally regulates ATF6 and ER to nucleus signaling 1 (*Ern1*), encoding IRE1, was found to play important role in regulation of plasma cell differentiation and antibody production as well (110). Different from XBP1s, IRE1α regulates both differentiation and antibody production in B cells. The phosphorylation of IRE1α triggers the splicing of XBP1 under lipopolysaccharide stimulation and further induces the antibody production in plasma cells (111). IRE1α also plays an important role in B-cell lymphopoiesis, which is required for Ig gene rearrangement and production of B cell receptors. IRE1α-deficient B cells were unable to develop beyond the pro-B-cell stage (79).

In addition, ER stress regulates production of proinflammatory cytokine by activating B cells. B cell priming and activation by antigen is associated with increased ER-stress-related gene expressions including GADD34, GRP78, and CHOP. Induction of ER stress by Tg treatment upregulates proinflammatory cytokine gene expressions (e.g., IL-23p19, IL-6, TNFα, IL-2, etc.) in B cells (112). These observations indicate the potential role of ER stress in B-cell differentiation and function in normal conditions and in diseases (Table 1).

### Dendritic Cell

DCs, which serve as professional antigen-presenting cells, are critical for the initiation of an adaptive immune response, and DCs are tightly regulated by ER stress (113, 114). XBP1-deficient lymphoid chimeras possess decreased numbers of both conventional and plasmacytoid DCs in mice, a phenotype that could be rescued by XBP1s overexpression in the hematopoietic progenitors. The expression of XBP1s is increased during the maturation of DCs in response to Toll-like receptor (TLR) signaling (113). In combination with ER stress, TLR stimulation enhanced binding of CHOP to the promoter of IL-23, thereby increasing IL-23 p19 production (83). XBP1s can also promote IL-23 production via mitochondrial ROS, in situations when excess fatty acid accumulates in the immediate milieu, resulting in impaired glycolysis (115). DCs stimulated by poly I:C also increase their production of IFNγ and inflammatory cytokines in a XBP1-dependent manner (116).

In the TME, the infiltrating DCs undergo profound ER stress, which compromises antitumor immunity. Targeting XBP1s in DCs enhances the antitumor immunity in several models. The Glimcher group has demonstrated that DC-specific deletion of XBP1s or selective nanoparticle-mediated XBP1s silencing in tumor-associated DC (tDC) increased T-cell-mediated antitumor immunity. This study also showed that XBP1s elevate the triglyceride biosynthetic program in tDCs, which led to the abnormal lipid accumulation and diminished antigen presentation. Genetically and pharmacologically targeting XBP1s in DCs converts tolerogenic DCs into immunogenic DCs (14). In the allogeneic bone marrow transplant setting, the inhibition of XBP1s reduced targeted organ damage and pathogenic Th1 and Th17 development without impacting donor Tregs or antitumor CTL. These findings were explained as a result of impaired generation of monocyte-derived DCs, leading to decreased alloactivation of T cells (117). Interestingly, recent work showed that IRE1α/XBP1s axis favors the cross-presentation of antigens from DCs to CD8^+^ T cells. DCs also play an important role in brain immunity. DCs are located in choroid plexus, pia mater, and dura mater, but not in the perivascular space of brain. These suggest that DCs may play a major role in recruiting T cells into the brain area. However, specific roles of UPR branches in brain residing DCs need to be further investigated (118). Pharmacological inhibition of IRE1α endonuclease function selectively blocks cross-presentation of tumor-associated antigen to major histocompatibility complex class I pathway without impairing presentation of tumor antigens to major histocompatibility complex class II, leading to inhibition of CD8^+^ T-cell priming (119). These contrasting observations draw attention to the potential pitfalls in connecting ER stress in DCs to diseases (Table 1).

### Macrophages

Macrophage is a crucial cell type involved in innate immunity which largely functions through phagocytosing pathogens and producing inflammatory cytokines. The polarization of macrophages is important for their function in producing pro- or anti-inflammatory cytokines (120). Specific ablation of IRE1α induces an M1–M2 imbalance in metabolic diseases. Abrogation of IRE1α in obesity promotes M2 polarization while limiting M1 polarization in a RNase-dependent manner. In cystic fibrosis patients, M2 polarization is defective, and IRE1α-XBP1 pathway induces mitochondrial metabolism in M1 macrophage and further promotes the inflammatory cytokine production (121). Despite regulating obesity and the cystic fibrosis, the roles of macrophage in liver diseases are closely linked to ER stress. STAT1 and STAT6 pathways are involved in the inhibition of M1 polarization and the promotion of M2 polarization in NAFLD (84, 122). As expected, knockdown of PERK alters STAT1 and STAT6 pathway in macrophage to increase NAFLD (86). As mentioned above, ER stress can also be induced by TLR activation. IRE1α-XBP1 pathway, but not PERK and ATF6, is positively regulated by TLR2 and TLR4. In addition, induced XBP1 expression promotes the proinflammatory cytokine (IL-6, TNFα, and IFNβ) production in macrophages (123, 124). Different from XBP1, ATF6 regulates the production of proinflammatory cytokines in macrophages through modulating TLR signaling, but ATF6 was generally not affected by TLR signaling (87). Moreover, knockdown of ATF6 in macrophages specifically decreased TNFα and IL-6 expression by limiting the activity of nuclear factor kappa B. Overall, targeting ER stress as a means to repolarize M2 macrophages in the TME may be an enticing therapeutic approach.

### MDSCs

MDSCs are a group of immune-suppressive cells in TME with heterogeneous phenotypes and functions. Same as other myeloid populations, ER stress plays important roles in regulating functions of MDSCs in cancer. Multiple administrations of low-dose Tg can cause ER stress and enhance immunosuppressive capacity of tumor-infiltrating MDSCs by upregulating arginase-1, inducible nitric oxide synthase, and NADPH oxidase 2 production, leading collectively to impairment of tumor-residing CD8^+^ T cell cytotoxic function (12). Furthermore, CHOP in MDSCs is a positive regulator for their suppressive function. ROS and nitrogen monoxide (NO) production in MDSCs are decreased in the CHOP-deficient MDSCs when compared to WT controls, which lead to lower IL-6 expression and STAT3 phosphorylation, suggesting the critical cancer-extrinsic role of CHOP (13).

### Other Immune Cells

Natural killer (NK) cells are critical controller of host immunological homeostasis and pathogenesis. Glimcher group showed that IRE1–XBP1 pathway mediates NK cell proliferation through direct regulation of c-Myc. In addition, XBP1 promotes the oxidative phosphorylation of NK cells (90). Our group have shown that platelet is a crucial mediator in antitumor immunity (125). The formation of platelets is mediated by ER stress. Using ER stressor, caspase-4 was inhibited in the megakaryocytes, which enhanced platelets production (91). However, the detailed mechanism is still not clear. Overall, the roles of ER stress in NK cells and platelets remain elusive (Table 1).

## UPR-Targeted Pharmacological Strategies to Balance Immune Response

**Figure 4:**
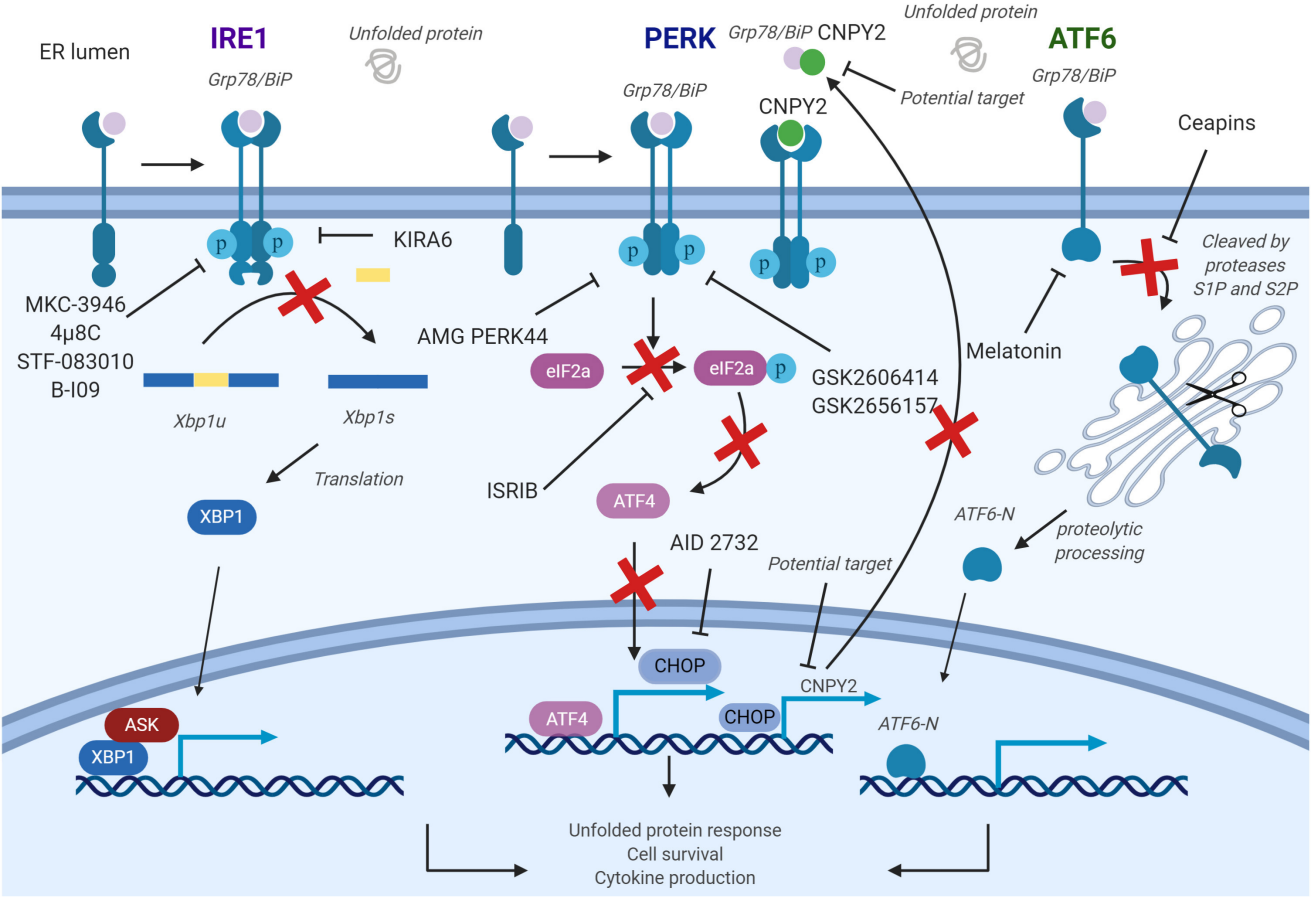
Pharmacological strategies to control endoplasmic reticulum (ER) stress in diseases. The inositol-requiring enzyme 1a (IRE1a) kinase domain can be directly inhibited by small molecule drugs, such as 4μ8c, MKC-3946, and B-I09. These compounds prevent splicing of the Xbp1 messenger RNA (mRNA). STF-08310, a compound selectively inhibiting ER stress-initiated endonuclease activity of IRE1, also prevents further downstream signaling. These compounds could have effect on T cells, B cells, dendritic cells (DCs), macrophages, myeloid-derived suppressor cell (MDSCs), and natural killer (NK) cells. PKR-like ER kinase (PERK) inhibitors are developed to inhibit the enhancement of PERK, and its downstream factors and could be used in targeting T cells, B cells, DCs, and macrophages. In addition, a CHOP-specific inhibitor was developed to prevent the CHOP promotor activation. Melatonin has also been reported to selectively inhibit ATF6. PERK–CHOP pathway could also be blocked by canopy homolog 2 (CNPY2) inhibition. Figure was made with Biorender.

Given the role of prolonged ER stress in multiple chronic medical conditions, considerable efforts have been made to develop small molecules that can reduce ER stress. In line with this consideration, inhibitors of ER stress signaling components including IRE1α, XBP1, PERK, ATF6, and CHOP has been developed. However, most of the agents have not been tested for their immunological properties particularly in relevant clinical models *in vivo*. Here, we summarize the mechanisms and current use of these UPR component inhibitors (Table 2, Figure 4) and call for future investigation of their use in immune modulations.

**Table 2:** Preclinical usage of unfolded protein response (UPR) inhibitors.

**Targets**	**Name**	**Mechanism**	**Current status**
IRE1α	MKC-3946	Binds to the endoribonuclease domain of IRE1α and inhibits its activity	Blocks MM tumor growth preclinical model (126). Induces apoptosis and G1 cell cycle arrest in acute myeloid leukemia cells (127).
	4μ8C	Covalently targeting IRE1 Lys907 via Schiff base formation	Inhibits MM cells growth *in vitro* (128). Blocks IL-5 production in Th2 cells (74). Inhibits the production of β-catenin from colon cancer cells (129).
	STF-083010	Selectively inhibits ER stress-initiated endonuclease activity of IRE1	Blocks MM tumor growth (130). Protects liver from fibrosis (131). Results in increased lysosomes and reduced viability of PDAC cells (132). Suppresses M2 phenotype through mediate IL4/IL13 pathway (133).
	KIRA6	Targets IRE1α kinase domain in order to allosterically disrupt endoribonuclease function of IRE1α in promoting XBP1 mRNA splicing.	Rescue genetically modified diabetic mouse model (Akita) from hyperglycemia and was able to protect destruction of pancreatic β cells resulting in increased production of insulin (134).
	B-I09	Inhibit IRE1 RNase activity	Disrupt IRE1–XBP1 pathway and prevent human CLL cells growth *in vitro* (135).
PERK	GSK2606414	Targets PERK in its inactive conformation at the ATP-binding region	Prevents pancreatic tumor growth (136). Increases MMP-2 production and inflammation (137).
	GSK2656157	Prevents ER stress-induced enhancement of PERK and eIF2α phosphorylation as well as ATF4 & CHOP upregulation	Inhibits M1 macrophage polarization (86). Decreases the angiogenesis ability of myofibroblast in liver fibrosis (138). Enhances glucose-stimulated insulin secretion (139).
	AMG PERK44	AMG PERK 44 does not show inhibitory effect against RIPK1, and it shows 160 times stronger specificity for PERK when compared to 300+ tested kinases.	PERK inhibition by these small molecules induced pancreatic β-cell toxicity, similar to what has been seen in the PERK knockout mouse, indicating more sophisticated methods to deliver these inhibitors are required (140).
ATF6	Melatonin	Selectively block ATF6	Sensitizes human hepatoma cells to ER stress inducing apoptosis (141).
	Ceapins	Trap ATF6, thus preventing translocation of ATF6 from ER to Golgi upon initiation of ER stress	Ceapins do not affect other arms of ER stress response such as IRE1 and PERK and can sensitize cells to ER stress without affecting normal cell function (142).
eIF2α	IRSIB	Reverse the phosphorylation of eIF2α	Prevents formation of stress granules exclusively triggered by eIF2α phosphorylation (143).
CHOP	AID 2732	Inhibitors of ER stress-induced CHOP promoter activation	High-throughput screening has been used to discover pharmacologic inhibitors of CHOP (144).

### IRE1α Inhibitors

Two classes of small molecule inhibitors for IRE1α has been developed as a means to control ER-stress-mediated diseases. The first group of IRE1α inhibitors bind to the RNase domain, which include MKC-3946, 4μ8C, STF-083010, and toyocamycin to name a few (145). MKC-3946 is a potent and soluble IRE1α inhibitor, and it has been proven for its ability in treating multiple myeloma in the preclinical models. MKC-3946 induces modest growth inhibition without toxic effect in normal cells; however, in combination with conventionally used drugs, MKC-3946 significantly induces cancer cell death (126). MKC-3946 also shows cytotoxicity against acute myeloid leukemia by inducing cell cycle arrest (127). 4μ8C is another selective inhibitor for IRE1α RNase domain. 4μ8C forms a stable imine bond at catalytic core of the RNase domain and blocks cleavage of XBP1 mRNA (145). 4μ8C-treated Th2 cell line shows reduced IL-5 production due to protein secretion defect, suggesting possible uses of 4μ8C in chronic inflammatory disorders (74). In colon cancer model, 4μ8C treatment inhibits cancer cell proliferation while reducing β-catenin expression (129). STF-083010 shows inhibitory effects in multiple myeloma xenografts mouse model. STF-083010 shows preferential cytotoxic effect to freshly isolated multiple myeloma cells when compared to other isolated immune cell populations such as B cells or NK cells (130). In addition, IRE1α inhibition by STF-083010 was able to increase lysosomes and show cytotoxicity against pancreatic ductal adenocarcinoma (132). In liver, STF-083010 treatment alleviates carbon-tetrachloride-induced liver damage and fibrosis (131). Toyocamycin also markedly inhibits ER stress in multiple myeloma cells, resulting in potent cytotoxic effect (146). Second class inhibitor, such as KIRA6, targets IRE1α kinase domain to allosterically disrupt endoribonuclease function of IRE1α in promoting XBP1 mRNA splicing. KIRA6 treatment was able to rescue genetically modified diabetic mouse model from hyperglycemia and was able to protect destruction of pancreatic β cells resulting in increased production of insulin (134). Another IRE1 RNase inhibitor called B-I09 can disrupt IRE1–XBP1 pathway and prevent human CLL cell growth *in vitro* (135).

### PERK Inhibitors

Biochemical screenings identified GSK2606414 and GSK2656157 as competitive PERK inhibitors by inhibiting PERK's interactions with ATP (136, 147). GSK2606414 has been shown to inhibit growth of pancreatic cancer cells while also restoring MMP-2 protein accumulation suppressed by ER stress in JEG-3 cells (137, 148). To increase pharmacological stability, GSK2656157 was later developed as a modified PERK inhibitor from GSK2606414. GSK2656157 treatment can inhibit M1-type macrophage polarization induced by ER stress and also increase glucose-stimulated insulin secretion (86, 149). Despite promising therapeutic effects, off-target issues have been reported for both inhibitors. GSK2606414 and GSK2656157 were identified as potent RIPK1 inhibitor, at a nanomolar concentration range, suggesting that beneficial effects of PERK inhibitors may not be accomplished solely by targeting PERK. To overcome these undesirable effects, AMG PERK 44 was identified. AMG PERK 44 does not show inhibitory effect against RIPK1, and it shows 160 times stronger specificity for PERK when compared to 300+ tested kinases (140). PERK inhibition by these small molecules induced pancreatic β-cell toxicity, similar to what has been seen in the PERK knockout mouse, indicating more sophisticated methods to deliver these inhibitors are required. Moreover, downstream pathway molecules are also the potential targets for ER stress. Integrated stress response inhibitor reverses eIF2α phosphorylation to disrupt ATF4 transcription and further regulates ER stress pathways (143).

### ATF6 Inhibitors

Development of an ATF6 inhibitor has been challenging, thanks in large part to the lack of druggable binding sites as well as minimal information in regards to its protein structure (150). However, using cell-based high-throughput assay, Ceapins were identified as potent ATF6 inhibitor (142). Ceapins are classified as pyrazole amides, and multiple studies suggest that Ceapins trap ATF6, thus preventing translocation of ATF6 from ER to Golgi upon initiation of ER stress (151). Ceapins do not affect other arms of ER stress response such as IRE1 and PERK and can sensitize cells to ER stress without affecting normal cell function (142). Melatonin has also been identified as novel selective ATF6 inhibitor. Melatonin selectively blocks ATF6 and further reduce cyclooxygenease 2 expression. This inhibition resulted in enhanced liver cancer cell death under ER stress (141). Based on therapeutic effects of Ceapins in ER-stress-related diseases, further optimization of Ceapins is required.

## Conclusive Remarks

UPR is an essential checkpoint for the ER homeostasis and serves as a physiological sensor for the stress from the accumulation of misfolded proteins in the ER. Molecular signaling mechanisms including UPR sensors IRE1, PERK, and ATF6 have been elucidated, as well as their signaling cascades and major mediators such as XBP1s and CHOP. Over the decades, role of ER stress in diseases has been broadly revealed including obesity, autoimmune diseases, cancer, liver diseases, neurodegenerative diseases, and others. However, it remains incompletely understood how ER stress affects immunity and immune-related diseases at the organismal level via regulating specific immune cell populations. This question is complicated since the immune response is a multistage process and that ER stress response can promote cell survival as well as cell death depending on circumstances that affect the cell fate.

ER stress maintains the homeostasis of the organism by regulating both pro- and anti-inflammatory pathways (Figure 3). The interests are high in elucidating the interrelationship between immune cells and ER stress (Table 1). IRE1 pathway so far has received the most attention and is better studied. However, the role of PERK and ATF6 pathways in regulating multiple immune cell populations is relatively more obscure. More studies are also necessary to understand the cross-talk among each branch of the ER stress pathways in immune responses during both physiological and pathological conditions.

The TME is an instructive model to study stress and adaptation. TME, particularly in the solid tumor settings, creates hostile conditions for both the tumor cells and the host immune cells, which can be characterized by nutrient deprivation (8), hypoxia (9), acidic extracellular pH (7), and ROS accumulation (6). Whereas, cancer cells adapt well to these harsh conditions, increasing evidence demonstrate that TME provides immunosuppressive stress signals to rewire the host immune system for immune evasion (152). Importantly, all of these metabolic conditions in the TME are activators of ER stress (153). On the one hand, rapidly growing tumor cells including breast, lung, brain, colon, glioblastoma, and pancreatic cancer cells are known to turn on UPR (154), which creates a potential strategy for targeted therapy. The examples of this effort are numerous. For example, forced expression of dominant negative IRE1α or inhibition of XBP1 by RNA interference reduced blood vessel formation in a human tumor xenograft model (155, 156). Inhibition of PERK pathway promotes oxidative DNA damages and impairs tumor survival under hypoxic condition (157, 158). On the other hand, emerging roles of ER stress in negatively regulating host immune responses such as T-cell antitumor immunity are reported. Thus, targeting ER stress can potentially be therapeutic for cancer via both cancer-intrinsic and extrinsic pathways.

As summarized in Table 2, preclinical studies have demonstrated the potential of UPR targeting in regulating immune cells. However, none of these agents have progressed to clinical approval for human diseases. There are concerns that targeting ER stress systemically could cause significantly undesired side effects, primarily due to loss of the quality control for normal organ function. Moreover, there remains significant challenges on how to achieve a balance between beneficial and harmful effects of UPR inhibition and if cell type-specific targeting of ER stress can be accomplished. For example, in light of the emerging role of ER stress in controlling immune cell activation, differentiation, function, and exhaustion in the TME, it is plausible that targeting ER stress in effector cells, such as T cells, is an efficient way to enhance immunotherapy. As such, a better understanding of precise ER stress pathways in various subsets of T cells (including memory T cells and exhausted T cells) is required. Considering the critical role of T-cell exhaustion in regulation of tumor growth and clearance, it requires a better understanding of T-cell exhaustion. ER stress has been linked to T-cell exhaustion by inducing the expression of inhibitory molecules such as PD1, TIM3, and LAG3 on the cell surface (65). Recent researches showed that the transcription factor TOX is required for the formation of terminally exhausted CD8^+^ T cells via chromatin remodeling and RNA transcriptome regulation (159–163). ER stress is an important factor that regulates chromatin changes in the tumor (164), as well as in the regulation of transcriptome (165). Therefore, ER stress may play previously unexplored roles in regulating TOX-mediated CD8^+^ T-cell exhaustion. Overall, the roles of ER stress in balancing immunity and tolerance in health and diseases are just beginning to be appreciated. Future work in this area is promised to be fruitful in the development of new immunotherapeutics.

## Author Contributions

AL and N-JS designed and drafted the work. All authors reviewed and critically edited the manuscript.

### Conflict of Interest

ZL serves as member of scientific advisory board for Alphamab and Henlius Biotech and has a sponsored research agreement with Bristol-Myers Squibb. The remaining authors declare that the research was conducted in the absence of any commercial or financial relationships that could be construed as a potential conflict of interest.
